#  Uterine closure with unlocked suture in cesarean section: Safety and Quality

**DOI:** 10.12669/pjms.303.4545

**Published:** 2014

**Authors:** Guluzar Arzu Turan, Esra Bahar Gur, Sumeyra Tatar, Ayse Gokduman, Serkan Guclu

**Affiliations:** 1 Guluzar Arzu Turan, MD, Department of Obstetrics and Gynecology, Sifa University Hospital, Izmir, Turkey.; 2 Esra Bahar Gur, MD, Sifa University Hospital, Izmir, Turkey.; 3 Sumeyra Tatar, MD, Department of Obstetrics and Gynecology, Sifa University Hospital, Izmir, Turkey.; 4 Ayse Gokduman, MD, Department of Clinical Biochemistry, Sifa University Hospital, Izmir, Turkey.; 5 Serkan Guclu, MD, Department of Obstetrics and Gynecology, Sifa University Hospital, Izmir, Turkey.

**Keywords:** Suture Techniques, Cesarean section, Ultrasonography, Niche, Surgical Techniques

## Abstract

***Objective:*** Comparing locked and unlocked uterine closure techniques in terms of bleeding control and uterine incision healing.

***Methods:*** The patients undergoing cesarean section in Sifa University Hospital between May - October 2012 were accepted to this prospective controlled study. Primarily, safety was evaluated. The hemoglobin count (HC) and serum creatine kinase (CK) levels of the patients in the locked (n = 47) and unlocked (n = 35) groups were measured just before and 24 hours after operation. Hemoglobin deficit, increase in CK and the additional hemostatic sutures were compared. Secondly, uterine scar healing was evaluated three months later. Scar thickness, niche and percentage of thinning of the scar region of the locked (n = 27) and unlocked (n = 32) groups were calculated and compared.

***Results:*** The hemoglobin deficit was similar in two groups. CK rise was less in the unlocked group but it was not significant (P = 0.082). Unlocked group needed more additional sutures (P = 0.016). The thickness of the niche and the percentage of thinning of the scar region were significantly less in the unlocked group (P= 0.002, P=0.000).

***Conclusions:*** Unlocked uterine closure technique is safe and has less damage to the myometrium.

## INTRODUCTION

 Cesarean section is one of the most frequent surgical procedures, in our country and around the world. Placentation abnormalities (such as placenta previa, placenta accreta, increata, percreata and uterine scar pregnancy) and separation or rupture of scar are the cesarean-related complications that may be faced with subsequent pregnancies.

 The technique in cesarean section has undergone many changes since it was first identified. Uterine repair is the most controversial issue. Double layer closure techniques were compared with single layer closure in 2008 Cochrane review. Single-layer closure was more advantageous in terms of blood loss during the operation. However, sufficient information was absent to decide the appropriate surgical technique.^1^

In a multicenter case-control study, the patients undergoing a trial of labor after a cesarean section were evaluated in terms of uterine rupture and uterine closure method. Two-fold increase in the risk of uterine rupture was reported to be related with single layer closure.^2^

 On the contrary, the meta-analysis published in 2011 revealed that locked but not unlocked single-layer closures were associated with a higher uterine rupture risk than double-layer closure in women at temping a trial of labor.^3^ These findings support Jelsema’ s hypothesis that locked suture technique may develop ischemic necrosis of tissue due to the increased pressure.^4^ However, recently the locked single-layer closure method of the lower uterine segment incision has been often preferred by surgeons to provide hemostasis.

Cesarean section scars can be identified reliably by trans vaginal ultrasound imaging.^5^^-^^7^ The association between the scar thickness and uterine rupture risk during trial of labor was also reported.^8^^,^^9^

However, we could not find any study defining the relationship between the scar image and suture technique. Therefore, we aimed to compare the locked and unlocked single layer closure of uterus in terms of perioperative safety (hemostasis) and post-operative quality (scar image).

## METHODS

 The study protocol was approved by the Ethics Committee of the Sifa University, Turkey. The prospective controlled study was performed after the approval between May 2012- October 2012 in accordance with the Helsinki Declaration. Patients who were planned for cesarean section were informed about the procedures that are likely to be applied. All participants were included in the study after informed consent. Three surgeons were responsible for the planned operations as one of them performed unlocked method. There was not a real randomization yet patients chose their surgeon without knowing which method would be applied.

 The main characteristic of the inclusion criteria of the study was that women who had reported medical indication for cesarean could be included in this study. Exclusion criteria were: previous uterine surgery (loop electrosurgical excision procedure, conisation, curettage, myomectomy), bleeding diathesis, arterial blood pressure higher than 150/90mmHg and the irregular separation of myometrium during the operation.

Demographic data (age, body mass index), gravidity, parity, primary or repeat cesarean characteristics were recorded .Study was performed in two stages: Perioperative safety primarily and evaluating the postoperative healing of the uterus secondly.

***Stage 1: ***There were 47 women in locked group and 35 women in unlocked group. Just before and 24 hours after operation blood tests were done on the patients. Hemoglobin Counts (gm/dl) were measured and the differences were calculated to detect the amount of blood loss. Serum creatine kinase (CK) levels were also measured. The increasing amounts of serum creatine kinase (CK) would give an idea about the destruction of the myometrial tissue.^10^^,^^11^ Hemoglobin Counts (gm/dl) and serum CK (U/L) were studied in the biochemistry laboratory (Hb: Rochesysmex; CK: Roche/Hitachicobas c 501 autoanalyzer, photometrically). And the duration of the operation and status of additional hemostatic sutures were recorded.

**Statistics:** SPSS 15.0 version was used to perform statistical calculations. Age, body mass index, gravidity, parity, primary or repeat cesarean characteristics and additional hemostatic sutures were compared with chi-square test. Variables such as operation time, hemoglobin deficit, increase in CK showed a normal distribution when analyzed. Parametric t test was used therefore.

**Fig.1:**
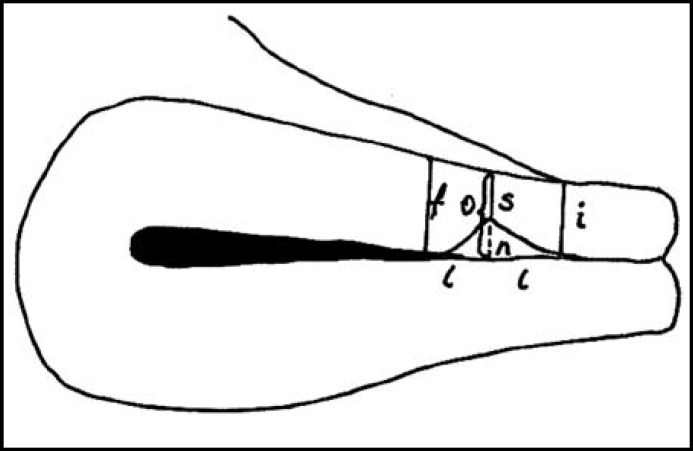
Scar thickness (s), myometrial thickness of the isthmus uteri at the level of the internal cervica los (i), the distance between the scar and the internal cervica los (l) and the myometrial thickness of fundal neighborhood of scar with interval of scar-isthmus distance (f). (o = (i + f) / 2, n = o – s).

**Fig.2:**
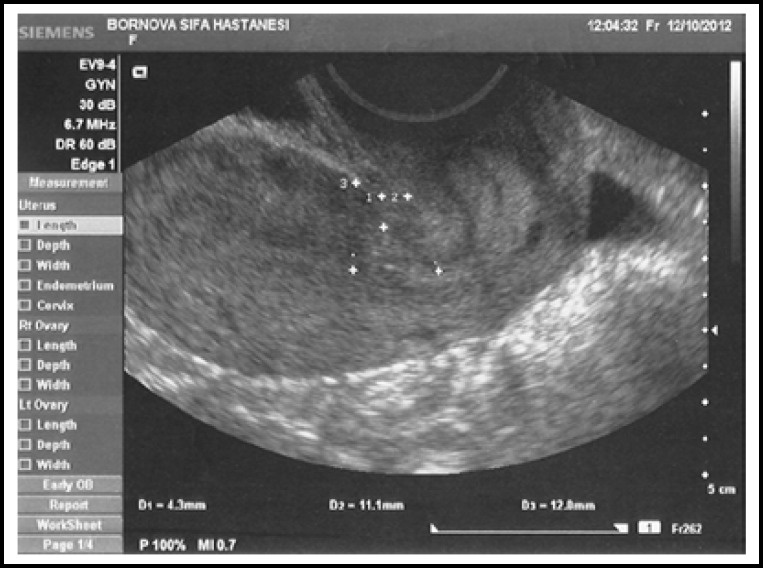
Ultrasound image of a uterus with locked method.

**Fig.3:**
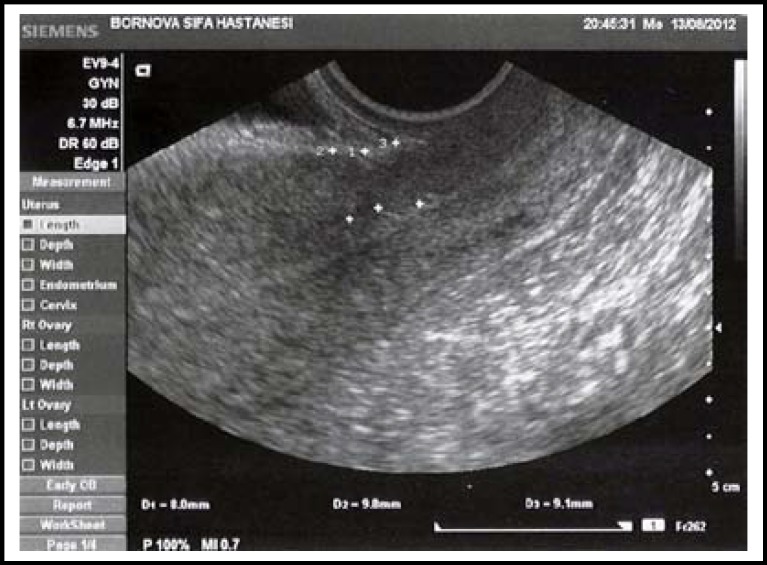
Ultrasound image of a uterus with unlocked method

***Stage 2: ***The patients were invited through phone calls to hospital for examination three months after operation. Patients with repeated cesarean section were not included in this stage. There were 27 women in locked group and 32 women in unlocked group. All of the patients were examined by the first author. Ultrasound examination was performed at dorsal lithotomy position with Siemens Acuson X300 EV 9-4 transvaginally after emptying the bladder. Anteverted or retroverted position of the uterus was recorded. The scar was determined in the sagittal plane. The following measurements were performed: scar thickness (s), myometrial thickness of the isthmus uteri at the level of the internal cervica los (i), the distance between the scar and the internal cervica los^l^ and the myometrial thickness of fundal neighborhood of scar with interval of scar-isthmus distance (f) (Fig. 1, 2, 3).

To eliminate individual differences, the Osser’s technique^5^ was modified and applied in this study. The average myometrial thicknesses of both sides of scar was assumed to be the original thickness of scar region (o).The scar thickness (s) was removed from the average thickness (o) to find scar thinning in this region (niche=n); (o = (i + f) / 2, n=o – s). In addition to this, the percentage of the thinning of the scar region was calculated (n / o).

**Statistics:** Age, body mass index, gravidity, parity characteristics were compared with chi-square test. Since values of the scar thickness, fundal neighborhood myometrial thickness, niche thickness and the percentage of thinning of the scar region were distributed normally, parametric t test was used.

## RESULTS

In the first stage, age, body mass index, gravidity, parity, primary cesarean rate were similar in the two groups. Hemoglobin deficit was also not different. CK rise of the unlocked group was less than the locked group but the difference was not statistically significant (*p = 0.082*). The number of patients needed additional suture was significantly higher in the unlocked group (17%vs.2%, *p= 0.016*) (Table-I). 

**Table-I:** Background Data and Perioperative Outcome (stage 1).

	*Locked (n = 47)*	*Unlocked (n = 35)*
Age (year)	28.4 ± 4.8	30.2 ± 5.3
Body Mass Index (kg/m²)	29.7 ± 4.2	30.6 ± 4.6
Primary cesarean	25 (53)	15 (43)
Patients additional hemostatic suture used	1 (2)	6 (17) *
Duration of operation (dk)	20.5 ± 2.5	21.1 ± 1.8
Hemoglobin deficit (gm/dl)	1.09 ± 0.75	1.10 ± 0.70
CK increase (U/L)	304.1 ± 258,6	203.7 ± 252 †

Data are n(%) or mean ±SD,

* p=0.016 (95%CI:0.028-0.271), †p=0.083 (95%CI:-13.25489- 214.22704)

**Table-II:** Background Data (stage 2).

*Parameters*	*Locked (n = 27)*	*Unlocked (n = 32)*
Age (year)	27.1 ± 5.1	28.1 ± 4.9
Body Mass Index (kg/m²)	29.8 ± 3.4	30.6 ± 6.6
Retrovert uterus (n)	8 (30)	10 (31)

Data are n(%) or mean ±SD

**Table-III:** Postoperative Ultrasound Findings (stage 2).

*Parameters*	*Locked (n = 27)*	*Unlocked (n = 32) *	*P values*	* 95 % CI*
Scar thickness (mm)	7.5±3.0	8.8 ±2.2	0.057	-0.04 ─ 2.64
Fundal region thickness(mm)	12.9±3.3	12.8±2.8	0.89	-1.70 ─ 1.48
Istmic region thickness (mm)	11.4±3.3	10.6±2.5	0.30	-2.30 ─ 0.72
Niche thickness (mm)	4.7±2.4	2.9±1.7	0.002*	-2.83 ─ -0.67
Thinning percentage (%)	38.4±17.3	24.3±12.7	0.000*	0.26 ─ 0.35

 In the second stage both locked and unlocked groups were similar in terms of age, BMI, gravidity, parity and retroverted uterus (Table-II). Although the scar was thicker in the unlocked group the difference was not significant (*p = 0.057*). However, the thickness of niche and thinning percentage was significantly lower in the unlocked group (*p = 0.002, p = 0.000*) (Table-III).

## DISCUSSION

Increasing rates of cesarean section cause concern so that poor results may occur in the subsequent pregnancies due to cesarean section. The subsequent attempts of trial of labor are the efforts to reduce the prevalence of cesarean section. Uterine rupture is the main problem that may appear in this order. The incidence of uterine rupture is 0.5– 4%.^12^ Differences in uterine closure techniques may affect the scar tissue durability due to tissue attenuation. Locked technique is the most commonly used method and the main reason for the lock is to control the bleeding. However, the tightening movement during locking may cause myometrial tissue necrosis. 

Rodrigues et al. measured the effect of suturing on ten different tissues such as fallopian tube, uterus, intestine and fascia. They mentioned that the gold standard was to manipulate tissue as gently as possible.^13^ Gul et al. made​​ an experimental study of the sheep, the individuals whose uterus left open and closed during a cesarean section were compared histopathologically. They observed myometrial necrosis in 100% of sutured group and 13.3% of the group left open.^14^

In 1993, Jelsema et al. compared unlocked suture with locked suture in the uterine closure in terms of security and control of bleeding. They stated that there was no difference. They also argued the hypothesis that unlocked method resulted in less tissue damage and a stronger wound theoretically.^4^ Over time, this relationship between the method and uterine rupture has been studied^4^^,^^15^, but there have not been any studies comparing the thickness of the scar.

In our study, in addition to improve the security of the unlocked method, we also sought to assess the healing of the scar. Similar blood loss showed adequate hemostasis. However it was observed that more additional suturing was needed (17%vs.2%). Some research shows that rising level of CK occurs as a result of a muscle tissue damage. Additionally, cesarean section and vaginal birth are known to increase CK levels.^16^^,^^17^ Also the destruction of the myometrial tissue causes serum CK increase.^10^^,^^11^

 Therefore, we measured CK levels to investigate if the locked method gave injury to the uterine muscle. The increase in the levels of CK in locked method was remarkable. Yet it was not statistically significant. The reason might be the release of CK from other damaged tissues in addition to uterus during cesarean section.

Transvaginal ultrasound has often been used in the evaluation of cesarean section scars. Myometrial thickness decrease was observed in patients who had undergone cesarean section, also scar dehiscence was observed in some patients by using transvaginal ultrasound.^5^^-^^7^ Hebisch et al compared the effectiveness of ultrasound and magnetic resonance (MR) imaging in the evaluation of the scar tissue. MR imaging was insufficient and ultrasound was better in the evaluation of the front wall of uterus.^18^ Regnard et al tried a different method, sonohysterography and showed the uterine scar dehiscence more clearly.^19^ Because it was more invasive than transvaginal ultrasonography, patients in this study were evaluated with tranvaginal ultrasound. The waiting period of three months after the operation was sufficient for resorption of sutures and the completion of the inflammation process.^20^

Ultrasonographic evaluation of the thickness of the scar may seem enough. However ranging sizes of uterus due to individual differences can result in error. Therefore, "niche" was calculated to eliminate individual differences. In this way, the amount of thinning of the wall thickness was evaluated. According to the results of stage 2 in our study, the amount of thinning of scar tissue in the myometrium was significantly lower in unlocked group. In other words, unlocked method has less damage to the myometrium. If we had evaluated only the thickness of the scar, unlocked group would have still been more advantageous. However, results were not significant due to limited number of the participants.

There were limitations in our study. We did not make power analysis before the study as we could not find a similar study. There was no real randomization and the study was not blinded.

 In conclusion, scar formation is unavoidable due to cesarean section. But we can review the suture techniques in order to minimize it. In clinical practice, we should be kind and respectful to the tissue as possible, because all forms of manipulation and dissection cause tissue reaction.

## Notes

***Funding: ***No specific funding was obtained.
